# Secondary bacterial infections of Carbapenem-Resistant *Acinetobacter baumannii* in patients with COVID-19 admitted to Chinese ICUs

**DOI:** 10.1186/s12866-025-04032-1

**Published:** 2025-05-22

**Authors:** Fuhong Chen, Jia Lin, Wei Yang, Jie Chen, Xiang Qian, Tao Yan, Xiuping Liu, Yewei Lu, Qi Chen

**Affiliations:** 1 Department of Clinical Laboratory, Hangzhou Traditional Chinese Medicine Hospital Affiliated to Zhejiang Chinese Medical University, Hangzhou, 310000 China; 2 Medical Intensive Care Unit, Hangzhou Traditional Chinese Medicine Hospital Affiliated to Zhejiang Chinese Medical University, Hangzhou, 310000 China; 3 Key Laboratory of Precision Medicine in Diagnosis and Monitoring Research of Zhejiang Province, Hangzhou, Zhejiang 310020 China

**Keywords:** Secondary bacterial infections, *Acinetobacter baumannii*, COVID-19, Carbapenem resistance, Whole genome sequencing, OXA-23

## Abstract

**Background:**

A significant proportion of patients who are hospitalized with coronavirus disease 2019 (COVID-19), particularly those being admitted to ICUs, exhibit the development of secondary bacterial infections (SBIs). However, there is a lack of detailed epidemiological investigations and genetic information of Carbapenem-Resistant *Acinetobacter baumannii* (CRAB) based on whole genome sequencing (WGS), which is one of the frequently detected bacteria among COVID-19 patients, to confirm alterations in the clonal structure and infection mechanism.

**Results:**

A total of 37 unique CRAB strains, sourced from patients, along with an additional 2 CRAB strains form the environment, were isolated. Among the cohort of 37 patients, 22 individuals succumbed to CRAB infection, resulting in a mortality rate of 54.46%. The median duration of illness for these patients was 7.95 days, highlighting the severity and rapid progression of CRAB infections in this patient population. A total of 22 CRAB strains, isolated from deceased individuals, in addition to two strains isolated from the environment, were subjected to further investigation. All 24 CRAB isolates exhibited a high ability to form biofilm and displayed a similar spectrum of resistance. Except for two isolates from patients with COVID-19, all the remaining CRAB isolates were categorized as ST195 and demonstrated highly close genetic background based on analysis of WGS. The ST195 strain of CRAB harbored three copies of the *bla*_OXA-23_ gene located on the chromosome, each of which was carried by Tn*2006*. Notably, one Tn*2006* element was integrated within Tn*6022*, leading to the formation of AbaR4-like resistance islands Tn*6166*-I.

**Conclusions:**

Our findings underscore the significance of SBIs in the COVID-19 pandemic, particularly those caused by CRAB and specifically those belonging to MLST types that were previously prevalent in ICUs.

**Supplementary Information:**

The online version contains supplementary material available at 10.1186/s12866-025-04032-1.

## Background

Since its initial emergence in December 2019 in Wuhan, China, Coronavirus disease 2019 (COVID-19), caused by the severe acute respiratory syndrome coronavirus 2 (SARS-CoV-2), has progressed into a global pandemic. According to data provided by the World Health Organization (WHO), by the end of 2023, the number of confirmed cases of COVID-19 has exceeded 772 million people, with global deaths surpassing 7 million (https://www.who.int/emergencies/diseases/novel-coronavirus-2019/situation-reports). The illness is typically of mild or moderate severity, manifesting as upper respiratory tract infections characterized by symptoms such as cough, runny nose, sore throat, fatigue, and chilliness, but occasionally could be turned into severe respiratory failure, particularly among older patients and those with underlying health conditions [1].

Secondary bacterial infections (SBIs) frequently develop in patients hospitalized with COVID-19, particularly in those who require invasive mechanical ventilation and are admitted to intensive care units (ICUs) [2]. Previous studies have consistently demonstrated that the incidence rates of SBIs among COVID-19 patients exhibit substantial global variation, with reported rates differing significantly across different regions and populations [3–5]. However, up to 50% of non-surviving COVID-19 patients experienced SBIs, which played an important role in the development of infections [6, 7].

*Acinetobacter baumannii*, an opportunistic pathogen, is consistently associated with respiratory tract infections. Multiple reports in the literature have documented secondary infections with *A. baumannii* during the COVID-19 pandemic [3–5]. In the Iranian ICU, a total of 19 COVID-19 patients were examined, with 17 found to be coinfected with *A. baumannii*. It was worth noting that this bacterium demonstrated a significant resistance to all antibiotics tested, with the exception of colistin. Regrettably, none of these patients who had this coinfection were able to survive [8]. During the initial wave of the COVID-19 outbreak in Wuhan, China, 102 patients acquired secondary bacterial infections, with 35.8% testing positive for *A. baumannii*. Notably, 91.2% of these infections were attributed to Carbapenem-Resistant *A. baumannii* (CRAB) [9]. The incidence of secondary infection caused by *A. baumannii* was reported to be as high as 18.1% (21/116) among hospitalized COVID-19 patients in ICUs of hospitals in Beijing between January 30, 2020, and April 13, 2020 [3]. Subsequently, China implemented rigorous prevention and control measures, leading to a reduction in confirmed COVID-19 cases to fewer than ten million by December 7, 2022. Nevertheless, by February 4, 2023, this figure had escalated tenfold following the relaxation of the stringent prevention measures. In this study, we conducted a systematic analysis of SBIs caused by CRAB among COVID-19 patients in the ICUs of a tertiary hospital in Eastern China during this specific period. The primary objective of this analysis was to identify the underlying mechanisms responsible for the hypervirulence of CRAB strains and highlight the significant role of secondary bacterial infections in COVID-19.

## Methods

### Information of the patients

Between December 7, 2022, and February 4, 2023, patients with severe pneumonia symptoms were admitted to two shared-staff ICUs without isolation rooms in a Hangzhou hospital. During this period, patients were housed separately but overlapped in the ICUs. Bacterial strains were isolated from specimens at the hospital's Clinical Laboratory. Permission was granted by patients'families, and the clinical characteristics of 37 patients were reviewed. Details like COVID-19/CRAB detection dates, ICU stay duration, devices used, sample types, and outcomes were extracted from hospitalization records.

### Bacterial strains

All isolates of *A. baumannii* initially from patients admitted to two ICUs were identified at the species level using matrix-assisted laser desorption/ionization time-of-flight mass spectrometry (VITEK**®** MS, BioMerieux, Marcyl'étoile, France). Bacterial stocks of each strain were stored at −80 ℃ in Brain Heart Infusion broth containing 20% glycerol (v/v). All the strains were thawed and sub-cultured in Columbia blood plate medium (Huankai Microbial, Guangzhou, China) for 18–24 h prior to use.

### Pulsed-Field Gel Electrophoresis (PFGE)

PFGE of *A. baumannii* was performed as described previously [10]. DNA from *Salmonella Choleraesuis* serotype Braenderup H9812 digested with *XbaI* (Takara, Beijing, China) was included as a molecular size marker. The fingerprints were analyzed with Denmark BioNumberics software version 7.6.1 (Applied Maths, St-Martenes-latem, Belgium) with a 1% optimization and a band-matching tolerance of 1%.

### Whole-genome sequencing

The cell biomass was harvested after 10 min centrifugation at 12,000 × g. DNA extraction was performed using the EZ-10 Spin Column Bacterial Genomic DNA Isolation Kit (Sangon Biotech, Shanghai, China) according to the manufacturer’s instructions. Purified genomic DNA was quantified by TBS-380 fluorometer (Turner BioSystems Inc., Sunnyvale, CA). High quality DNA (OD_260/280_ ≥ 1.5, ≥ 150 ng) was used to do further research.

The draft genome sequence analysis of CRAB strain was carried out using short reads by Illumina NovaSeq6000 sequencing platform (MajorBio Co., Shanghai, China) and long reads by PacBio Sequel II platform (Pacific Biosciences, Menlo Park, CA, USA) with the SMRT bell TM Template kit (Pacific Biosciences) according to the manufacturer’s instructions.

### Genomic analysis

The long (TGS) and short (NGS) WGS reads were trimmed with Filtlong v0.2.1 (https://github.com/rrwick/Filtlong) and fastp v0.23.2 [11], respectively. Genome assembly was performed based on trimmed reads using Unicycler v0.5.0 [12] with default settings.

Gene predictions and functional annotations were performed with PGAP [13]. The presence of acquired antibiotic resistance genes (ARGs) and chromosomal resistance mutations was detected with CARD database [14]. Virulence factors (VF) were screened using the VFDB database [15]. The search for insertion sequence (IS) elements and their characterization down to the family was carried out correspondingly using digIS v1.2 [16] based on ISfinder database [17]. Complete transposons (Tn) were detected using BacAnt v3.3.3 [18]. The structure of *A. baumannii* Resistance Island (AbaR) present in Z11-34 genome was visualized by gggenes (https://github.com/wilkox/gggenes).

Furthermore, a cgMLST allele calling was performed with chewBBACA suite v3.3.1 [19] using the Prodigal training file for *A. baumannii* from cgMLST.org (https://www.cgmlst.org/ncs/schema/Abaumannii1464/). Pairwise cgMLST distances were calculated with cgmlst-dists v0.4.0 (https://github.com/tseemann/cgmlst-dists).

### Antimicrobial Susceptibility Testing (AST)

Antimicrobial susceptibility testing was performed using the Vitek 2 Compact System (BioMerieux, Marcyl'étoile, France) to determine the minimal inhibition concentrations (MICs) for the following antibiotics: Ceftriaxone, Cefepime, Imipenem, Gentamicin, Tobramycin, Ciprofloxacin, Levofloxacin, Trimethoprim/sulfamethoxazole. MIC was determined using the E-test (Bio-kont, Wenzhou, China) method for Cefoperazone–sulbactam. The MICs of colistin and tigecycline were determined using the microbroth dilution method (Bio-kont, Wenzhou, China). The results were interpreted according to the Clinical and Laboratory Standard Institute guidelines 2022 except for tigecycline, which was assessed following the US Food and Drug Administration Approved break-points for Enterobacteriaceae (susceptible ≤ 2 mg/L; resistant ≥ 8 mg/L) [20]. The breakpoints for Enterobacteriaceae susceptibility and resistance to Cefoperazone–sulbactam were referred to the ones of Cefoperazone provided by CLSI, which are ≤ 16/4 mg/L and ≥ 64/4 mg/L, respectively. *Pseudomonas aeruginosa* ATCC 27853 and *E. coil* ATCC 25922 were used as the reference control strains.

### Biofilm assay

Biofilm formation was performed according to our previously described methods [9] with minor modifications. The absorbance was read at 570 nm using a Varioskan LUX microplate absorbance reader (Thermo Scientific, Waltham, MA, USA). In accordance with the criteria established by Stepannovic *et. al* [21], the cut-off value of optical density (OD_c_) was represented by 3 × SD above the mean values of the control wells. *A. baumannii* ATCC 19606 and ATCC 17978 were used as the negative and positive controls, respectively. The strains were classified into the following categories: 1) strong biofilm producer (OD > 4 OD_c_); 2) moderate biofilm producer (2 OD_c_ ≤ OD < 4 OD_c_); 3) weak biofilm producer (OD_c_ ≤ OD < 2 OD_c_); 4) no biofilm producer (OD ≤ OD_c_).

## Results

### Clinical characteristics

**Fig. 1:**
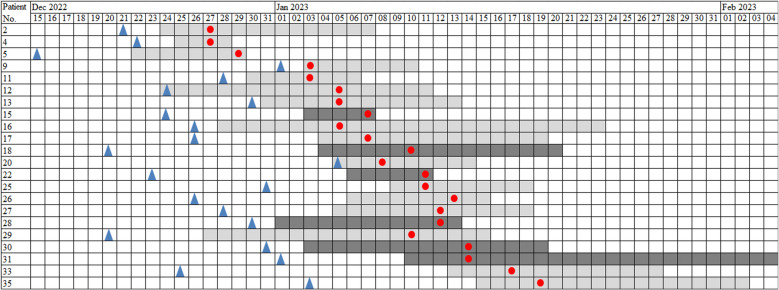
Timeline of the colonization or infection of the 22 decreased patients with CRAB.:  Date of the first SARS-COV-2;  Date of the first CRAB; light grey: duration of stay in ICU-A; dark grey: duration of stay in ICU-B

During the study period, a total of 37 patients were admitted to two ICUs in a tertiary hospital located in Hangzhou city. They were hospitalized due to SARS-CoV-2 pneumonia and subsequently developed SBIs caused by CRAB (the MIC of Imipenem ≥ 8 μg/ml). Ultimately, 22 patients passed away (Table 1). The mean age of patients was 82.86 years, with male patients predominating (77.27%). Almost all these patients presented with multiple comorbidities including hypertension, diabetes mellitus, heart disease, hyperlipemia and other chronic diseases. Moreover, mechanical ventilation was conducted in all the patients in the ICUs as a result of respiratory failure during admission. The median time to death after the occurrence of CRAB secondary infection was found to be 7.95 days. The events that took place throughout the inpatient admission period, including the time spent in ICUs and the isolation of *A. baumannii* strains, were illustrated in Fig. 1. CRAB strains were detected in samples procured from sputum, blood, and hydrothorax. A significant association appeared to be present between the presence of CRAB in the blood and the patient outcomes. Unfortunately, all four patients (No. 12, 13, 17, 20) did not survive after the detection of CRAB in their blood.

**Table 1:** Characteristics of patients and CRAB isolates from ICUs

Patient No	Isolate No	A, G	ICU	Admission diagnosis	Comorbidities^a^	Location of 1 st CRAB	Outcomes^b^	ST
1	Z11-15	73, F	A	Pneumonia	HD	Sputum	D	195
2	Z11-17	87, F	A	Pneumonia	DM	Sputum	D	195
3	Z11-18	88, M	A	Pneumonia	HT, CI	Sputum	D	195
4	Z11-24	88, M	A	Pneumonia	HD	Sputum	D	195
5	Z11-26	90, M	A	Pneumonia	CC, HD, HT	Sputum	D	195
6	Z11-27	53, M	A	Pneumonia	CKD, DM, HT	Sputum	D	195
7	Z11-28	94, F	A	Pneumonia	HD, HT, AD	Sputum	D	195
8	Z11-32	90, M	B	Pneumonia	DM, HT, CI	Sputum	D	195
9	Z11-33	87, M	A	Pneumonia	LD	Sputum	D	195
10	Z11-34	80, M	A	Pneumonia	MI	Sputum	D	195
11	Z11-35	67, F	B	Pneumonia	N	Sputum	D	208
12	Z11-40	98, M	A	Pneumonia	HD, AD, HT	Blood	D	195
13	Z11-42	90, M	B	Pneumonia	DM, HT	Blood	D	195
14	Z11-53	92, M	A	Pneumonia	CKD, HL	Sputum	D	195
15	Z11-58	81, M	A	Pneumonia	HT, LD	Sputum	D	208
16	Z11-60	90, F	A	Pneumonia	DM, HT, HD, HL	Sputum	D	195
17	Z11-61	97, M	B	Pneumonia	CC, HT, HD, CI, AD	Blood	D	195
18	Z11-62	67, M	A	Pneumonia	HT	Sputum^c^	D	195
19	Z11-63	89, M	B	Pneumonia	HT, HD	Sputum	D	195
20	Z11-65	65, M	B	Pneumonia	CKD, CI	Blood	D	195
21	Z11-72	85, M	A	Pneumonia	HT,	Sputum	D	195
22	Z11-78	72, M	A	Pneumonia	DM, CKD	Sputum	D	195
/	Z11-89	/	A	/	/	Bed handle	/	195
/	Z11-90	/	A	/	/	Vaporizers	/	195

^a^*HD* heart disease, *DM* diabetes mellitus, *CKD* chronic kidney disease, *HT* hypertension, *AD* Alzheimer's disease, *CI* cerebral infarction, *N* neoplasia, *CC* calculous cholecystitis, *LD* liver disease, *MI* myocardial injury, *HL* hyperlipemia

^b^*D* Decreased, *S* Survived

^c^CRAB was also detected in Hydrothorax

In this study, a total of 44 environmental samples were collected, including 30 samples collected from frequently touched surfaces, 8 samples taken from high-level surfaces that are difficult to touch, and 6 samples obtained from hand hygiene practices. Only two strains of CRAB were identified on the bed handle and on the surface of the vaporizers used by two recognized patients.

### PFGE and MLST

Initially, PFGE was employed to investigate the genetic relatedness among the 24 CRAB strains. The PFGE analysis, using *ApaI* digestion yielded the clustering of different strains into distinct groups when applying an 80% similarity threshold. Notably, a total of 21 isolates, originating from 20 patients and 1 environmental source, exhibited remarkably similar band patterns. Furthermore, a separate isolate (No. Z11-89) from the environment differed by only one band (Supplementary Figure 1), indicating a close relationship with the other isolates. Since the Oxford scheme, which involved the analysis of seven specific housekeeping genes (*cpn60*, *gdhB*, *gltA*, *gpi*, *gyrB*, *recA*, and *rpoD*), was known for its superior discriminatory power in distinguishing closely related isolates, we chose to use it for further investigations. Except No. Z11-35 and Z11-58, which had different pulsetypes but belonged to ST208 (equivalent to ST2 in Pasteur scheme), all strains were allocated to ST195 (equivalent to ST2 in Pasteur scheme). To elucidate the genetic relationships among various CRAB strains, whole-genome sequencing was performed on a comprehensive set comprising 22 strains isolated from deceased patients and 2 strains sourced from the environment.

### Whole-genome sequencing cgMLST analysis

**Fig. 2:**
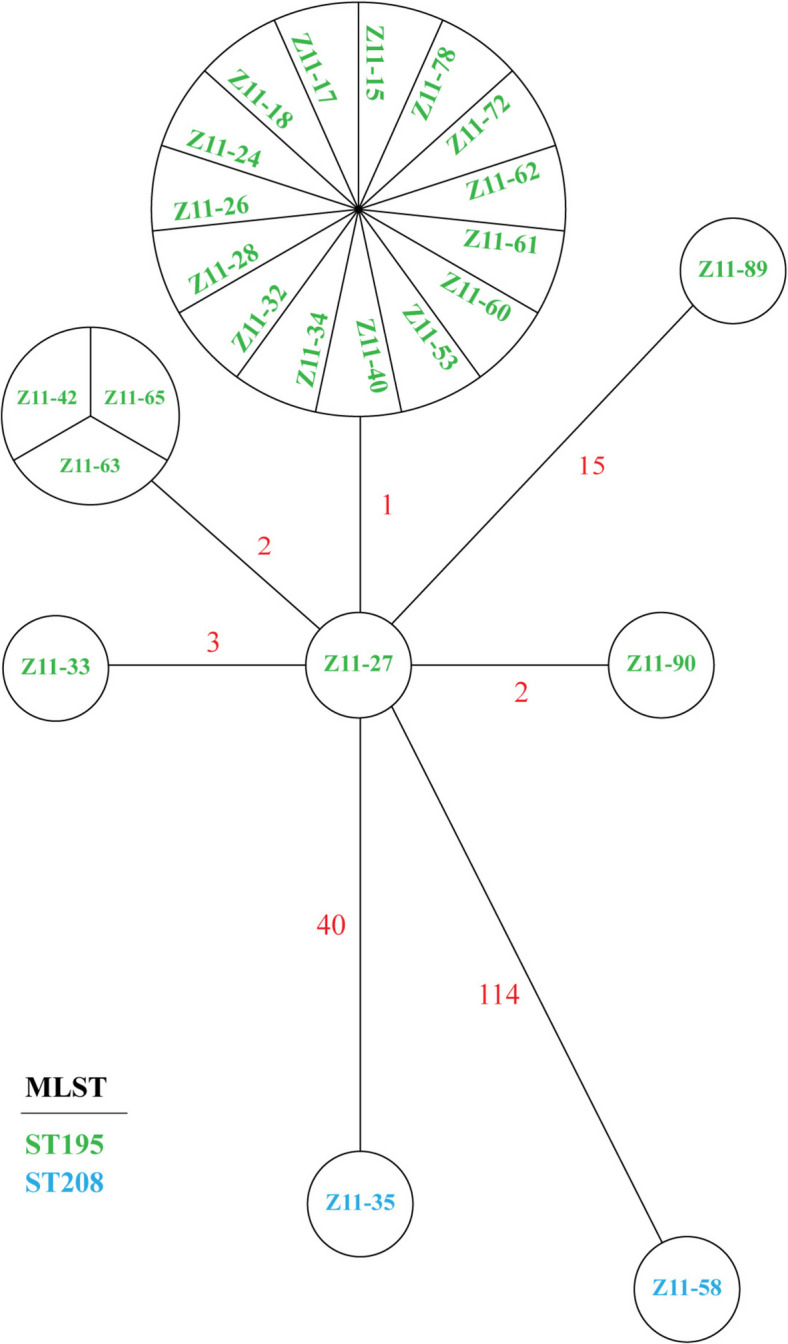
A minimum spanning tree was constructed utilizing the allelic genes of cgMLST from 24 CRAB strains isolated from ICUs. Each individual circle represents an allelic profile, which is derived from the sequential analysis of 2390 cgMLST genes. The lengths of the connecting lines indicate the quantity of target genes exhibiting diverse alleles

Whole-genome sequencing produced each of 24 *A. baumannii* strains raw data genome data of an average size of 1.27 GB. A high-resolution cgMLST analysis revealed that these 22 ST195 CRAB strains could be categorized into two distinct clades, exhibiting a genetic divergence of no more than 10 alleles (Fig. 2). The different variants among the ST195 isolates ranged from 0 to 15 alleles, indicating the close genetic homology between them. At random, isolate Z11-34 was chosen to be additionally sequencing using PacBio long-read sequencing technology.

### Susceptibility of CRAB strains to various antimicrobial agents

Antibiotic susceptibility testing was conducted on the 39 *A. baumannii* isolates after verifying their identity using VITEK**®** MS. All isolates demonstrated resistance to β-lactamases, including Ceftriaxone, Cefepime, Cefoperazone-sulbactam, and Imipenem, as well as aminoglycosides such as Gentamicin and Tobramycin. Additionally, resistance was observed against Quinolones like Ciprofloxacin and Levofloxacin, and Folate inhibitors such as Trimethoprim/sulfamethoxazole. Conversely, all strains of *A. baumannii* were found to be susceptible to Colistin B and Tigecycline, as indicated by the measured MIC values.

**Fig. 3:**
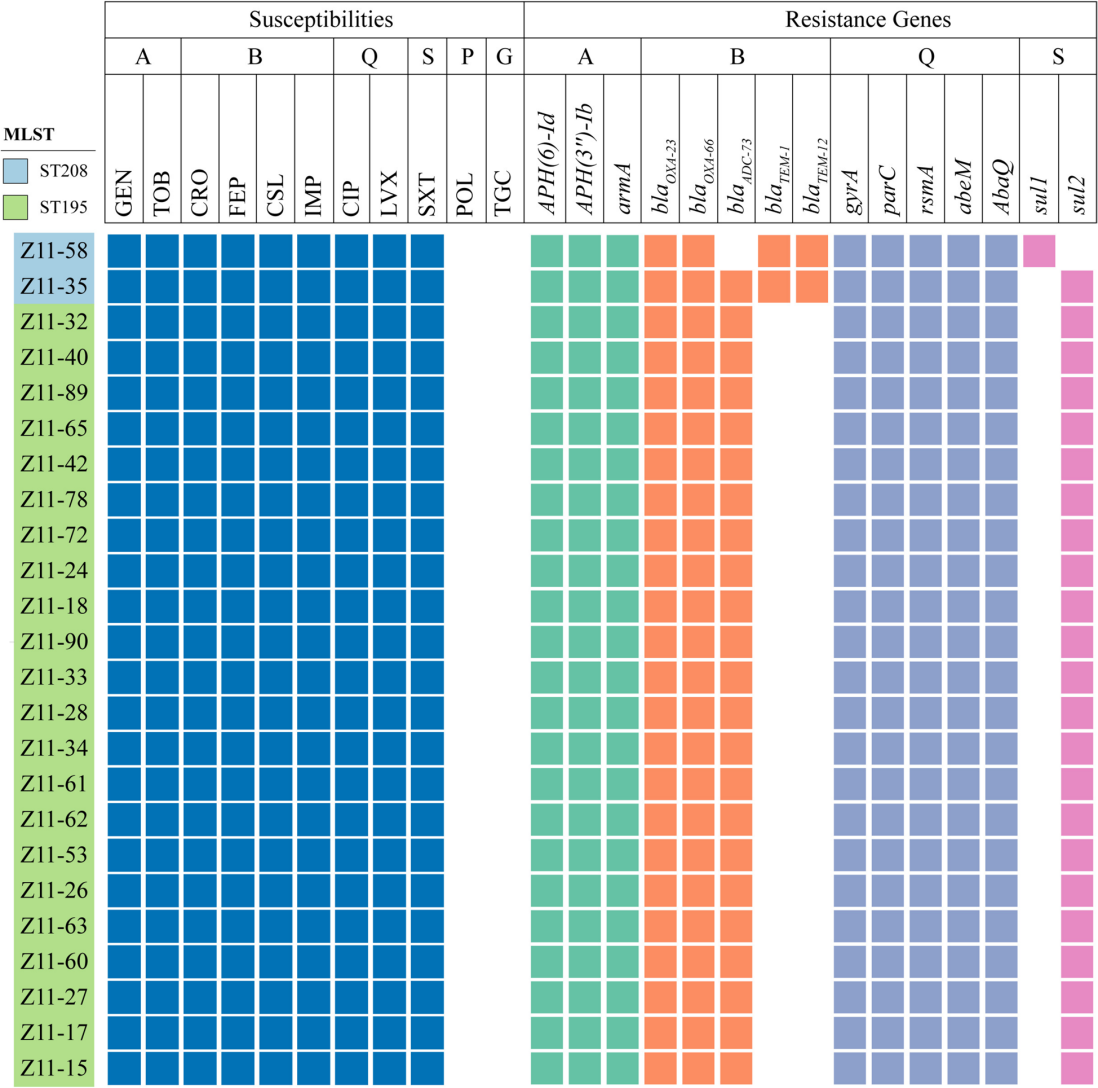
Distribution of resistance genes. Classes of antibiotics were given the following markings: A Aminoglycosides, B β-lactamases, Q Quinolones, S Sulfonamides, P Polymyxins, G Glycylcyclines, GEN Gentamicin, TOB Tobramycin, CRO Ceftriaxone, FEP Cefepime, CSL Cefoperazone–sulbactam, IMP Imipenem, CIP Ciprofloxacin, LEV Levofloxacin, SXT Trimethoprim/sulfamethoxazole, POL Colistin, TGC Tigecycline

The antibiotic susceptibility profiles of 24 CRAB strains were validated through WGS (Fig. 3). All isolates showed the presence of *Aminoglycoside phosphotransferase aph (3″)-lb* and *aph (6’)-ld*, and *armA*, indicating resistance to aminoglycosides antibiotics. Carbapenem resistance was attributed to the presence of *bla*_OXA-23_, the most frequently encountered Class D β-lactamases, in addition to *bla*_OXA-66_ and *bla*_ADC-73_. All CRAB strains tested positive for resistant genes linked to Quinolones (*gyrA*, *parC*, *rsmA, abeM* and *AbaQ*) as well as Sulfonamides (*sul1* or *sul2*). The resistance genes associated with the efflux pump resistance mechanism were also detected, namely *adeA*, *adeB*, *adeC*, *adeS*, *adeN*, *adeR*, *adeL*, *adeF*, *adeH*, *adeI*, *adeJ*, *adeK*, *AmvA*, *tet R*, *tet (B)*, and *AdaF*.

### Biofilm production assay

**Fig. 4:**
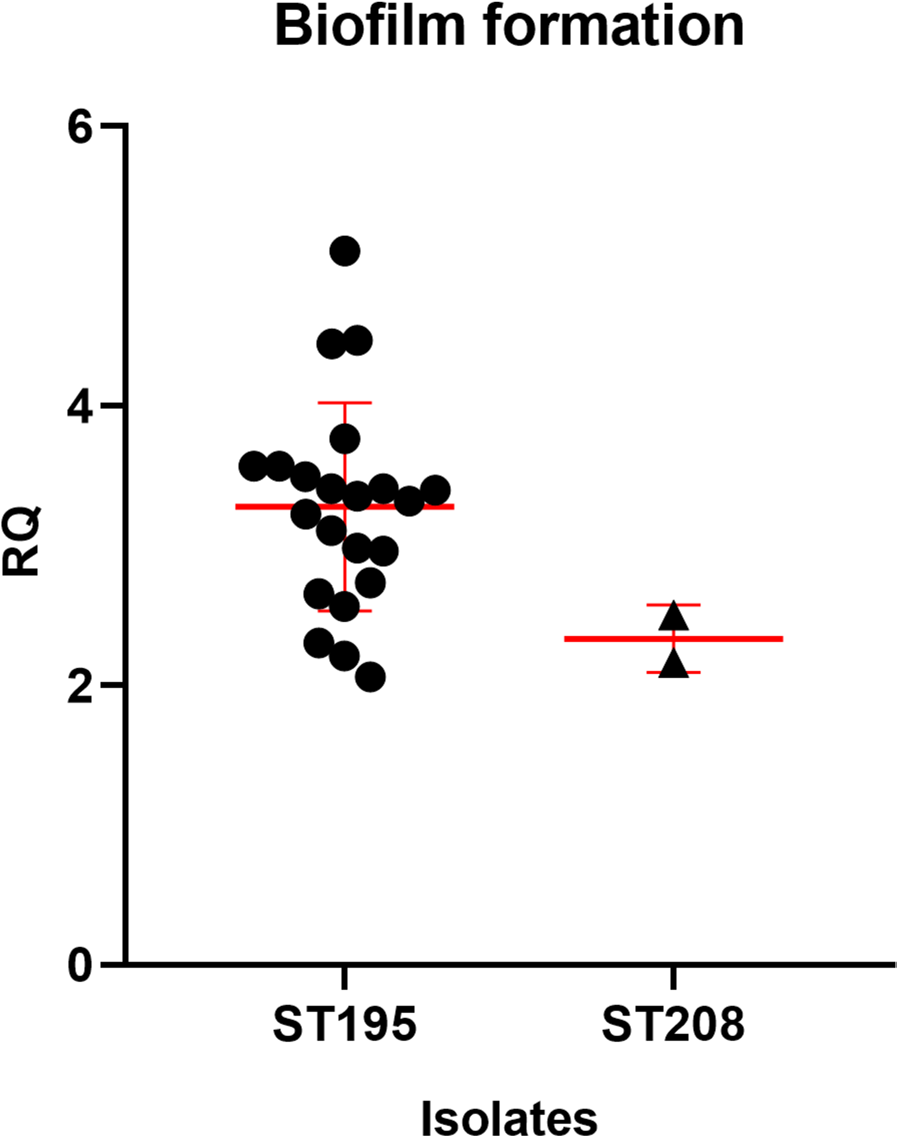
Biofilm formation of ST195 and ST208 CRAB isolates. The ordinate represented the ratio of each OD to the cut-off value of optical density (OD_c_)

**Fig. 5:**
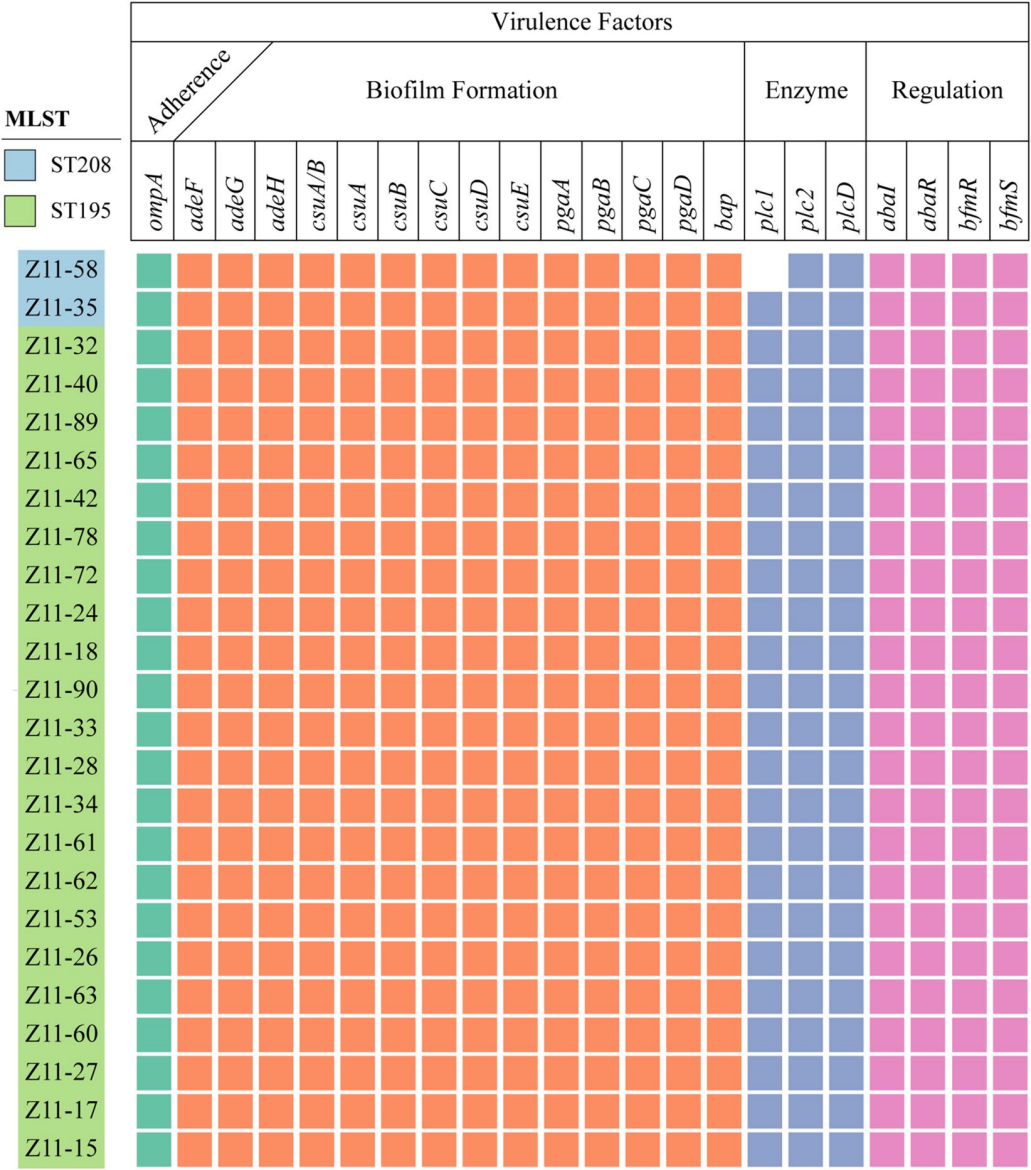
Distribution of antibiotic resistance and virulent genes

The ability to form biofilm, which possess the characteristics of persistence and resistance to eradication, is crucial for pathogenic bacteria in the progression of infections. For this purpose, the biofilm production of 24 isolates were conducted (Fig. 4). Regardless of the outcome of the patients, all ST195 isolates were classified as strong (*n* = 3) and moderate (*n* = 19) producers. The final two ST208 isolates exhibited lower efficiency in biofilm formation compared to the ST195 variants and were belonged to moderate producers. *bap*, whose encoding production plays a crucial role in biofilm formation and subsequent adherence to biotic surfaces, was present in all CRAB isolates in this study. Other factors suggested to modulate the formation of biofilm in *A. baumannii*, including PNAG genes (*pgaD*, *pgaC*, *pgaB*, *pgaA*), the quorum sensing system (*abaI* and *abaR*) and the biofilm-controlling response regulator (*bfmR/S*) were all identified in the genomes of the isolates (Fig. 5).

### Genetic elements of bla_OXA-23_ gene

**Fig. 6:**
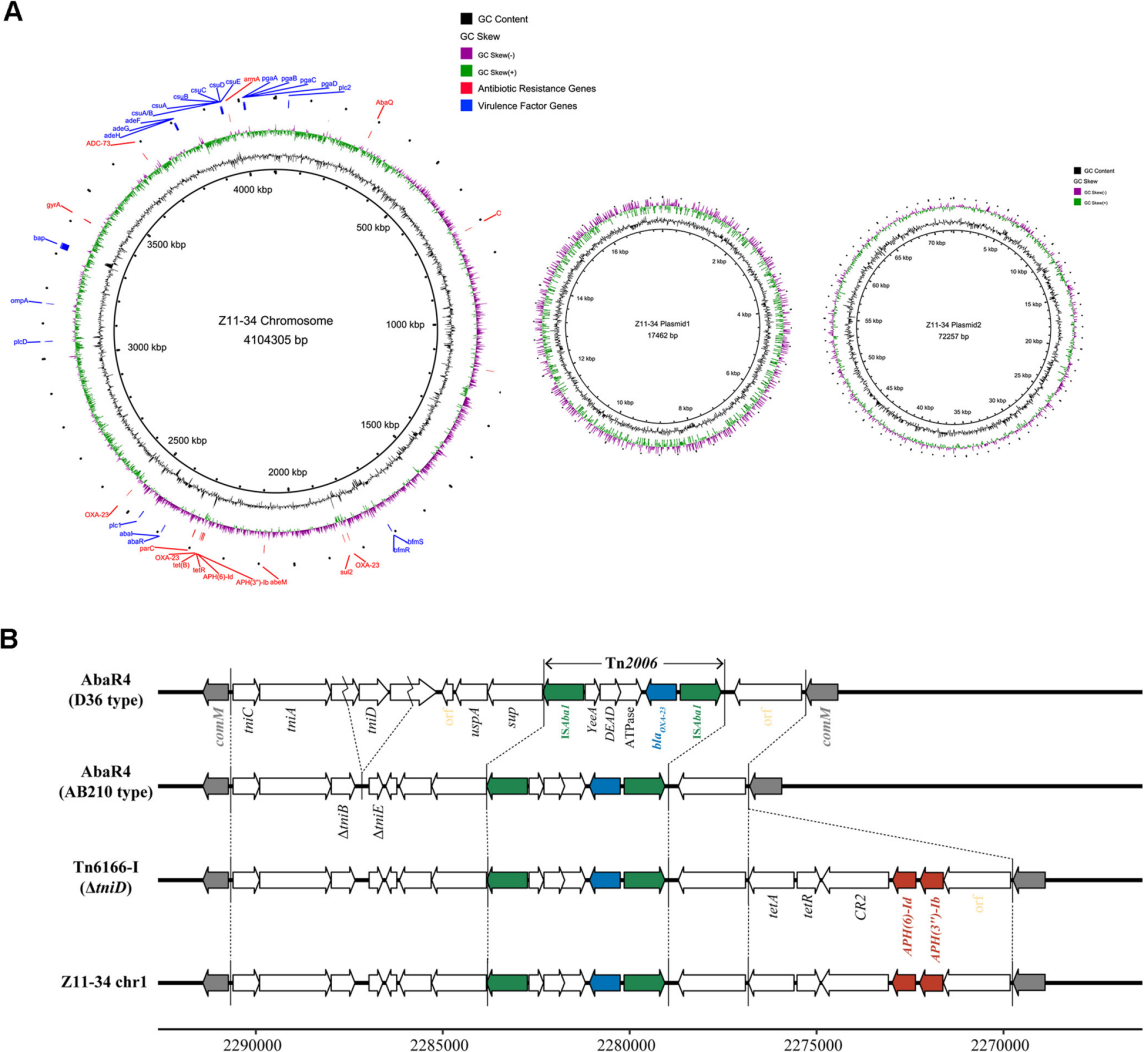
Analysis results of long-read sequencing technology. **A** Circular representation of the strain Z11-34 genomic sequence for the chromosome (Left panel) and the plasmids (Middle and Right panels). **B** Structures of AbaR4-like resistance islands. AbaR4-D36, AbaR4-AB210, *Tn*6166-I, and Z11-34 (this study) include *Tn*2006 with a *bla*_OXA-23_-like gene. In AbaR4-AB210, Tn*6166*-I, and Z11-34, a region including *tniD *and its upstream and downstream sequence was partially deleted

WGS of the isolate Z11-34 using NovaSeq6000 and PacBio Sequel platform provided a complete chromosome sequence (4,104,305 bp) and two plasmids sequence (17,462 bp and 72,257 bp) (shown in Fig. 6A). All the resistance genes were identified on the chromosome. Virulence factors including genes related to biofilm formation, adherence, enzyme and regulation as illustrated in Fig. 5, were also detected on the chromosome.

All ST-195 CRAB isolates from patients and the environment carried the *bla*_OXA-23_ carbapenems gene, in addition to intrinsically carrying *bla*_OXA-66_ gene. The *bla*_OXA-23_ is commonly found in the plasmids or integrated into the *A. baumannii* chromosome through transposons. To gain a comprehensive understanding of the mechanism driving the multiplication of *bla*_OXA-23_ in CRAB, our study focused on analyzing the types of transposons and the genomic location of *bla*_OXA-23_. This ST195 CRAB strain contained three copies of *bla*_OXA-23_ on the chromosome, each of which carried by Tn*2006*. Consistent with prior research, the Tn*2006* identified in this study consisted of two inversely oriented copies of IS*Aba*1 flanking an internal segment that contained the *bla*_OXA-23_ gene as well as *yeeA* (encoding the putative DNA methylase), *DEAD* (encoding the putative Asp-Glu-Ala-Asp helicase), and *ATPase* (encoding the putative AAA ATPase) genes (Fig. 6B). Subsequently, we examined the disparities among transposon insertion sites. One Tn*2006* was integrated within Tn*6022* resulted in the formation of AbaR4-like resistance islands Tn*6166*-I. Moreover, two additional Tn*2006* were found, which encoded for a hypothetical protein with an unknown function.

## Discussion

Herein, we conducted the first study to investigate the impact of secondary infections on COVID-19 patients who were admitted to ICUs for acute respiratory failure during the early phase of the pandemic. This phase occurred after the easing of strict prevention and control measures in China in late 2022. A total of 39 non-repetitive CRAB strains were isolated from patients and the ICU environment. Unfortunately, 22 out of 37 patients experienced a decline after contracting CRAB infection with a median duration of 7.95 days. Except for two isolates from COVID-19 patients, the remaining CRAB presented similar genetic backgrounds, including PFGE band patterns, MLST types, resistant virulent genes, and biofilm-forming abilities.

Coinfections, or secondary infections, have been shown to play an essential role in the high mortality rate observed among hospitalized patients with COVID-19 [7, 9, 22] and other respiratory viruses [23]. Influenzae, along with *Staphylococcus aureus* or *Streptococcus pneumoniae*, is commonly reported [24, 25]. During the 2009 H1 N1 pandemic, 71 of 838 children infected with HIN1 virus, have been diagnosed with *S. aureus* infection, of which 48% tested positive for MRSA [26]. Previous studies have indicated that SBIs occurred in approximately 10–15% of COVID-19 patients [2, 9], contributing to their higher mortality rate. A significant proportion of these patients necessitate invasive procedures, including tracheal intubation and mechanical ventilation. Similarly, all patients enrolled in our study were subjected to such procedures. *A. baumannii* is an opportunistic pathogen mainly linked to ventilator-associated pneumonia, especially the Carbapenems-Resistant *A. baumannii* [27]. During the COVID-19 pandemic all over the world, the higher incidence of *A. baumannii* infection (from 3.9% to 90%) have been reported multiple times in literature compared with other bacteria and fungi [2]. During the initial surge of COVID-19 epidemic, spanning from December 2019 to August 2020, a total of 102 COVID-19 patients (6.8%) who were admitted to hospitals in Wuhan, contracted secondary bacterial infections. This phenomenon was predominantly attributed to the presence of multi-resistant *A. baumannii* (35.8%), and tragically, nearly half of them (50 out of 102) passed away while being hospitalized [9]. Meanwhile, in a designated COVID-19 hospital located in Beijing, *A. baumannii* was detected in 20% of samples collected from the severely ill COVID-19 patients during the later stages of admission to ICU. This discovery was facilitated by the utilization of the RT-PCR assay technique [3]. The precise mechanisms governing this transmission should be subject to further investigation.

*A. baumannii* is commonly considered as a low-virulent pathogen, causing opportunistic infection in immunocompromised individuals. However, it can evolve to be fatal when the clone is referred to some special Sequence Type. *A. baumannii* strains LAC-4 (ST10) first isolated in Los Angels County in 1997, not only exhibited hypervirulence in immunocompetent mice [28], but also caused a fetal outbreak in China [29]. Another lineage closely related genetically to the LAC-4 strain, called MRSN 16897, presented hypervirulence and caused mortality in conventional, immunocompetent BALB/c mice [30]. Other unique Sequence Types, such as ST758 (strain AB030), ST457 (strain AB31) and ST945 (strain AB5075), were found to be Carbapenem-Resistant hypervirulent *A. baumannii* (CR-hvAB) strains using either mice models or *Galleria. mellonella larvae* infection models [31–33]. The ST195 CRAB strains, consistently identified as the predominant strain worldwide, have the potential to cause mortality in patients [34]. Ghiwa Makke, et al. reported that five out of six ST195 strains were significantly associated with patient mortality in ICUs [35]. Patients infected with ST195 exhibited higher 7-day and 28-day mortality rates compared to other ST groups, except for ST457 [33]. The *G. mellonella larvae* infected with a single ST195 strain, isolated from a critically ill hospitalized patient in the mid-south region of China, was without survival [32]. Given the absence of specific genetic markers for hypervirulence in CRAB strains, it will be necessary to utilize animal models infected with the ST195 strain in this study to further evaluate and assess its virulence potential.

*A. baumannii* is capable of forming biofilms on various types of surfaces, highlighting the vital role of the biofilm phenotype in infections [36]. We found that isolates of ST195 were more efficient in forming biofilm than ATCC 17978 and were categorized as strong or moderate biofilm producers. The genes *bap* and *ompA*, known to be essential for *A. baumannii* biofilm formation [37], were all captured in the genome of ST195 isolates. β−1,6-poly-N-acetyl-D-glucosamine (PNAG) is a surface polysaccharide that plays a vital role in maintaining biofilm integrity [38]. Genes involved in PNAG synthesis were identified in ST195 CRAB isolates (Fig. 5). A recent study has demonstrated that the Csu pili not only serve as essential components of mature *A. baumannii* biofilms but also play a significant role as a virulence factor facilitating bacterial adherence to epithelial cells [39]. These pili are encoded by an operon that consisted of *csuA/B, csuA, csuB, csuC, csuD* and *csuE*, Thus, the presence of the Csu pili operon in ST195 could be a primary factor contributing to the mortality of infected patients. However, the scarcity of antibodies specific to biofilm-related proteins in *A. baumannii* on the current market poses a significant challenge. This shortage hinders the ability to accurately discern the differences in the expression patterns of these proteins between the ST195 and ST208 strains.

Carbapenems were used to be the first-line treatment for multidrug-resistant *A. baumannii*. However, the past decade has seen an increasing occurrence of CRAB all around the world [40], which has resulted in treatment failure and prolonged hospitalization. The OXA-type carbapenem-hydrolyzing class D β-lactamase plays a crucial role in carbapenem resistance of *A. baumannii* [41]. Among these enzymes, *bla*_OXA-23_ is the most predominant carbapenems gene in CRAB and its dissemination is consistently associated with the presence of composite transposons. To date, four main types of transposons, namely Tn*2006*, Tn*2007*, Tn*2008*, Tn*2009* and several novel ones (Tn*7534* and Tn*6549*) have been found to mediated the transfer of *bla*_OXA-23_ in *A. baumannii* [42, 43]. Tn*2006* is the most frequently described transposon harboring *bla*_OXA-23_ [42], and it efficiently transfers genes between isolates through plasmid or itself. Previous research suggests that Tn*2006*, either located on the chromosome or the plasmids, is the predominant transposons carrying the *bla*_OXA-23_ gene in ST195 strains [44, 45]. Conversely, Tn*2009*-harboring isolates have typically been confined to ST208 strains [45]. In this study, three copies of the *bla*_OXA-23_ genes were found to be consistently co-located on the chromosome and carried by Tn*2006*. Future research should be conducted to examine the association between distinct transposons and the molecular epidemiology of *A. baumannii*, in relation to specific MLST types.

Different transposon insertions can have an impact on both upstream hypothetical proteins and potentially generate novel resistance genes at the special locus [44]. Previous studies have reported insertions by Tn*2006* upstream of ZnuA which a substrate-binding protein of the zinC-uptake ABC transporter, as well as flavin mononucleotide (FMN) reductase and transcriptional regulator CmtR [46]. However, the most common insertion site of Tn*2006* is the comM-AbaR, which is an antibiotic resistance island [47]. In this study, two copies of Tn*2006* were independently inserted upstream of unknown hypothetical proteins and the last one, identical to Tn*6166*-I, formed the subtype of AbaR4-like resistance islands (RIs). Previous investigations have demonstrated that AbaR4-like RIs, comprising AbaR4 (D36 type), AbaR4 (AB210 type), Tn*6166*-I and AbaR25-I (as illustrated in Fig. 4B), were found to be common among Asian isolates of CRAB [45, 48]. Notably, Tn*6166*-I has been identified as a component of CRAB CC92 strains (ST92 and ST395), which also encompass the ST195 lineage. However, another study also reported the discovery of two additional, novel variants of AbaR4-type RIs within the chromosomes of Malaysian ST195 strains [49]. This finding further emphasizes the extensive genetic diversity and variability observed in AbaR4 RIs in the context of the ST195 strain background.

## Conclusion

Secondary bacterial infections may occur during or following a COVID-19 infection, ultimately resulting in high mortality rates. *A. baumannii* strains with a high drug resistance spectrum are consistently identified as the primary bacteria. Owing to the severity of the pandemic conditions, it was impractical to establish a control group without COVID-19 in our ICUs simultaneously. Consequently, this limitation has introduced uncertainty in our understanding of the relationship between CRAB coinfection and the substantial mortality rate among our patient cohort. Hence, further investigation is recommended to corroborate this hypothesis. However, the spread of CRAB-ST195, which harbors three copies of *bla*_OXA-23_ gene conferring carbapenems-resistance, within ICUs, has become a significant concern. Consequently, it is crucial to implement precise measures for controlling nosocomial infections and monitoring the mortality rate among critically ill COVID-19 patients.

## Supplementary Information

Supplementary Material 1

## Data Availability

The whole genome sequencing data in this study have been deposited in GenBank under BioProject ID PRJNA1084207.
