# Supplementary material for: Secondary bacterial infections of Carbapenem-Resistant Acinetobacter baumannii in patients with COVID-19 admitted to Chinese ICUs
Source: BMC Microbiol. 2025 May 22;25:319. doi: 10.1186/s12866-025-04032-1 (PMC12096753; doi:10.1186/s12866-025-04032-1)
Supplement: Supplementary file 1 — Supplementary Material 1 [file 12866_2025_4032_MOESM1_ESM.docx]

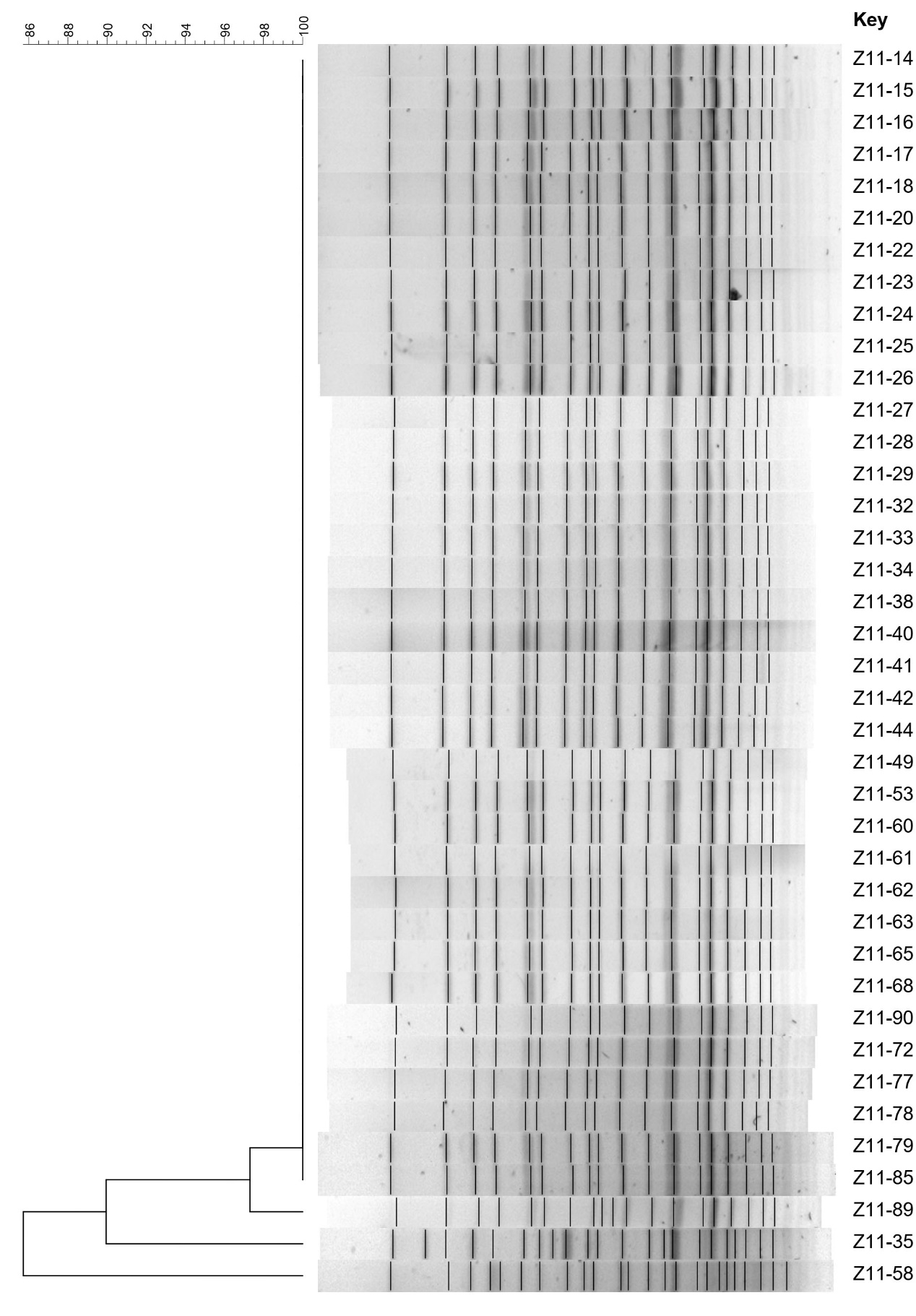


Supplementary Figure 1. Pulsed-field gel electrophoresis (PFGE) profiles of 24 *Acinetobacter baumannii* isolates from ICUs. The scale indicated percent similarity. Isolates were designated with the numbers given in Table 1.
